# How can transforming representation of mathematical entities help us employ more cognitive resources?

**DOI:** 10.3389/fpsyg.2023.1091678

**Published:** 2023-03-02

**Authors:** Omid Khatin-Zadeh, Danyal Farsani, Adriana Breda

**Affiliations:** ^1^School of Foreign Languages, University of Electronic Science and Technology of China, Chengdu, China; ^2^Norwegian University of Science and Technology, Trondheim, Norway; ^3^University of Barcelona, Barcelona, Spain

**Keywords:** representation, mathematical metaphor, motor system, motor strength, gesture

## Abstract

This article discusses the cognitive process of transforming one representation of mathematical entities into another representation. This process, which has been called *mathematical metaphor*, allows us to understand and embody a difficult-to-understand mathematical entity in terms of an easy-to-understand entity. When one representation of a mathematical entity is transformed into another representation, more cognitive resources such as the visual and motor systems can come into play to understand the target entity. Because of their nature, some curves, which are one group of visual representations, may have a great motor strength. It is suggested that directedness, straightness, length, and thinness are some possible features that determine degree of motor strength of a curve. Another possible factor that can determine motor strength of a curve is the strength of association between shape of the curve and past experiences of the observer (and her/his prior knowledge). If an individual has had the repetitive experience of observing objects moving along a certain curve, the shape of the curve may have a great motor strength for her/him. In fact, it can be said that some kind of metonymic relationship may be formed between the shapes of some curves and movement experiences.

## Introduction

A mathematical concept, idea, or problem may have a variety of representations. When we are faced with a mathematical problem, we may represent it in the form of a table, a diagram, a graph, words, algebraic symbols, numbers, or other possible channels of representation. Since mathematical entities and relations are inherently abstract, we operate on them through their representations (Selling, 2016). Throughout this paper, we use the expression *mathematical entities* to generally refer to mathematical concepts, ideas, or problems. For example, in abstract algebra, groups and operations defined among elements of groups can be represented through tables, diagrams, graphs, or algebraic symbols. Learning how to represent a mathematical entity and how to transform one representation into another representation in order to find the best way to solve a problem is one of the principal mathematical skills. Dreyfus (1990) suggests that the process of learning a multi-representational mathematical problem takes place in four stages: the first stage involves the formation of a single representation in the mind of the learner; in the second stage, the learner creates several parallel representations in her/his mind; in the third stage, the learner identifies the connections between various representations; the fourth stage involves the integration of representations and flexible movements between various representations. Using multiple mathematical representations is a way for enhancing mathematical creativity (Boaler et al., 2016; Bicer, 2021a,b) as it enables students to flexibly shift between various representations and alternate their solutions when they encounter new problems (San Giovanni et al., 2020). It has been suggested that applying multiple mathematical representations can help students figure out connections among various concepts and develop their mathematical knowledge (Ervynck, 1991; Silver, 1997; Levav-Waynberg and Leikin, 2012).

Representations are tools through which people construct mathematical ideas and think and communicate about them (Pickering, 1995; Singh, 1998; Bass, 2011). People can develop a deeper understanding of mathematical concepts and ideas by examining a variety of representations (Maher et al., 2010). Learning to work with parallel representations of the same idea or problem is a key factor in the process of mathematics learning (Selling, 2016). This has been supported by the findings of some empirical studies that have provided evidence showing how learning mathematics can be enhanced through working with multiple representations (e.g., Brenner et al., 1997; White and Pea, 2011; Boaler, 2015; Boaler et al., 2016; Selling, 2016; Bicer, 2021a,b). The following sections look at the ways through which transforming representations can help learners to employ their cognitive resources more effectively in the process of learning and the grounding of mathematical concepts into concrete environment.

## Transforming representations and embodiment

When one representation of a mathematical entity is transformed into another representation, the two representations share an underlying structural similarity. In other words, it is the superficial representation that is changed not its underlying conceptual structure. Therefore, a set of parallel representations of a mathematical entity are isomorphic with each other at an abstract level. That is, while they share a basic abstract structure, they seem to be different concretely. Based on Lakoff and Johnson’s (2003) definition of metaphor, transforming one representation of a mathematical entity into another representation is basically a mathematical metaphor (see also Pim, 1988). It is a metaphor because we structure, understand, and manipulate one representation in terms of another representation. According to Lakoff and Johnson (2003), the essence of metaphor is describing and understanding an unfamiliar abstract concept (target of the metaphor) in terms of a familiar concrete concept (base or source of the metaphor). The description of numbers in terms of points on a line and the description of functions in terms of curves in the Cartesian plane are two common mathematical metaphors that have been discussed by Lakoff and Núñez (2000). Mathematical metaphors are not inherently different from common linguistic metaphors. Both types of metaphor describe a concept in terms of another concept. Perhaps the only difference between mathematical metaphors and linguistic metaphors is that there is a mathematical logic behind every mathematical metaphor. This may make mathematical metaphors more rigorous and more precise than linguistic metaphors.

Mathematical metaphors allow us to embody an entity through another entity. From the perspective of the strong version of embodiment theories (Gallese and Lakoff, 2005), the same cognitive resources that are employed to understand the base (source) domain of a mathematical metaphor may also be employed to understand the target domain of that metaphor. The weak version of embodiment takes a broader view and holds that concepts are understood through a process in which sensory-motor, emotional, and modality-independent systems are involved (e.g., Binder and Desai, 2011; Kiefer and Pulvermüller, 2011; Meteyard et al., 2012; Hauk and Tschentscher, 2013; Lambon-Ralph, 2013; Zwaan, 2014; for reviews, see Tirado et al., 2018; Khatin-Zadeh et al., 2021). For example, the mathematical metaphor *f(x) oscillates between-1 and 1* describes a function in terms of the movement of an object that oscillates between the two extreme points of-1 and 1. The strong version of embodiment theories holds that the same cognitive resources that are employed during observing the oscillation of a moving object are also employed to process the mathematical metaphor *f(x) oscillates between-1 and 1*. Therefore, the strong version of embodiment theories predicts that processing this metaphor involves the activation of the motor system as this metaphor describes a function in terms of a motion domain (Lakoff and Núñez, 2000; see also Marghetis and Núñez, 2013). We will return to this point in more detail later.

**Figure 1:**
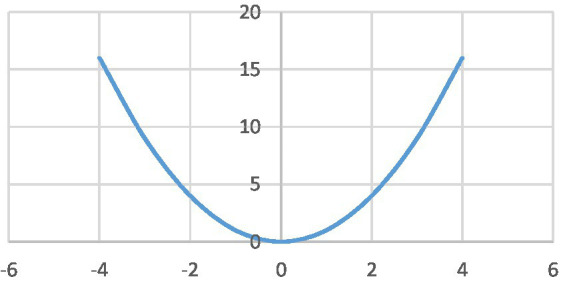
Visual representation of the function *f(x) = x*^2^ in the Cartesian coordinate system.

When one representation of a mathematical entity is transformed into another representation, new resources can come into play to understand the base entity (the first representation) in a more effective way (Khatin-Zadeh, 2022). An example is the transformation of abstract mathematical entities into visual representations such as graphs and diagrams. A curve that represents a mathematical function in the Cartesian coordinate system is one of the most common visual representations in mathematics. The standard symbolic representation of the function is in the form of *y = f(x)*. Transforming abstract mathematical entities into visual representations is a very common practice in learning mathematics and other fields of science. For example, the function *f(x) = x^2^* can be represented by a curve in the Cartesian coordinate system (Figure 1). The visual representation of this function is much easier to process than its standard symbolic representation. This is particularly the case with students at early stages of learning the concept of function.

**Figure 2:**
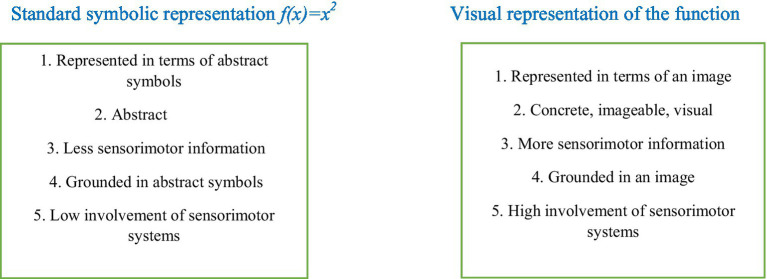
A one-to-one comparison between the features of standard symbolic representation of a function and its visual representation standard symbolic representation *f(x) = x*^2^ visual representation of the function.

Visual tools have a strong degree of imageability. Therefore, from the perspective of strong version of embodiment theories (Gallese and Lakoff, 2005), when an abstract mathematical entity is transformed into a visual representation and is understood in terms of that visual representation, that entity is grounded by the support of the visual system. In this way, the sensory-system is employed to ground an abstract entity into concrete environment. In Figure 2, a schematic diagram of changes that happen when the standard symbolic representation of *f(x) = x*^2^ is transformed into a visual representation in the Cartesian coordinate system. In this figure, a one-to-one comparison is made between the features of the two representations.

Another example is the transformation of arithmetic operations into finger-based representations. Some finger counting studies have provided evidence that supports this idea (e.g., Lindemann et al., 2011; Wasner et al., 2014; Barrocas et al., 2020). For example, Sato et al. (2007) used transcranial magnetic stimulation to examine excitability changes in hand muscles when a group of people were performing a visual parity judgment task. The results showed an increase in amplitude of motor-evoked potentials in right hand muscles. This supports the embodied representation of numbers and arithmetic operations in hands/fingers. In a recent study, Artemenko et al. (2022) used a finger-based method for teaching arithmetic operations to a group of children. After 1 year of training, this group of children showed finger-related arithmetic effects accompanied by activation in the sensorimotor cortex during performing mental arithmetic operations. This suggested that after training, children used finger-based numerical representations to perform mental arithmetic operations.

The extent to which the abstract entity can be grounded through sensory system depends on perceptual strength of the base entity. If the base entity has a high visual strength, the visual system is effectively employed to ground and understand the abstract entity. In recent years, a number of studies have examined degree of perceptual strength of concepts in a variety of languages (e.g., Filipović Đurđević et al., 2016; Speed and Majid, 2017; Miklashevsky, 2018; Chen et al., 2019). These studies have shown degree of visual strength or imageability of concepts varies across a broad range. While some concepts have a very low degree of visual strength, others have a very high degree of visual strength. Between these two extreme points of visual strength, concepts may have a wide range of visual strength. Those concepts which have a high degree of visual strength can effectively be used to ground and understand abstract concepts through the visual system. Since diagrams, graphs, tables, coordinate systems, and vectors have a high degree of visual strength, they are effective tools for grounding abstract mathematical entities. Here, the main job of the learner is to transform the abstract representation of a mathematical entity into a strongly visual representation. In this way, a wider range of cognitive and perceptual resources are employed to process the abstract mathematical entity.

A recent comprehensive study (Lynott et al., 2019) examined degree of perceptual and action strength of a large set of concepts. Results obtained in this study indicated that some concepts have a high degree of action or motor strength, while some other concepts have a very low degree of action or motor strength. Between these two extreme points, concepts may have a wide range of motor strength. Even words which do not directly refer to a motion event may have some degree of motor strength. This could have some implications for transforming one representation of a mathematical entity into another representation. When the abstract representation of a mathematical entity is transformed into a visual representation, the abstract representation is understood through a representation that has a high degree of visual strength. As mentioned, in this way, the visual system contributes to the process of grounding and understanding. Furthermore, the motor system may also come into play. Since even some non-motion concepts may have some degree of motor strength, the visual representations of abstract mathematical entities may have some degree of motor strength. If this is the case, it can be said that the motor system may play at least a partial role in the processing of abstract mathematical entities. A question that may be raised here is how it is possible for a static visual representation to have some degree of motor strength. Discussing several evidence from a number of past studies, the following two sections try to answer this question.

## Motor system activation during visual perception

Findings of some studies suggest that static pictorial stimuli showing implied motion can be the cause of motor system activation (e.g., Winawer et al., 2008; Williams and Wright, 2009; Osaka et al., 2010; Lorteije et al., 2011). Some neuroimaging studies have provided evidence suggesting cortical areas involved in real motion processing are activated by pictorial stimuli showing implied motion (e.g., Kourtzi and Kanwisher, 2000; Senior et al., 2000; Kim and Blake, 2007). Some studies have gone beyond this and have found evidence suggesting even visual stimuli that do not contain implied motion can be the cause of activation in the motor system. Among these studies, one group has specifically focused on visual perception of letters. It has been found that looking at static letters can activate cortical motor areas (Longcamp et al., 2003; James and Gauthier, 2006). Longcamp et al. (2011) compared the neural correlates of perceiving handwritten letters vs. printed letters. They found that visual perception of handwritten letters involves a stronger activation in left primary motor cortex and the supplementary motor area. The strong activation of motor areas during perceiving handwritten letters could be the result of simulating those hand actions that produce the handwritten letters. It has been shown that observing some static visual stimuli such as a cut in a canvas activates cortical motor system (Gallese and Sinigaglia, 2011). Umilta’ et al. (2012) examined the neural activities during observing static images of abstract paintings produced by Lucio Fontana. They found that cortical motor system is involved in the processing of static abstract art works.

**Figure 3:**
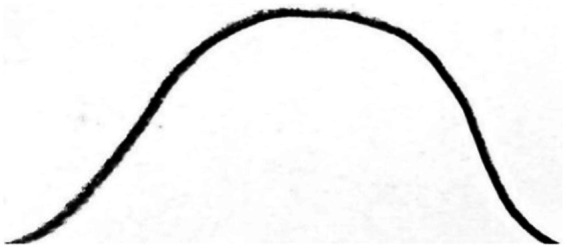
Hand-drawn graphical representation of the function *f(x) = − x*^2^.

If motor areas are activated during visual perception of handwritten letters and abstract art works, they may also be activated during processing a curve. In fact, if a simulation of hand movements takes place during observing handwritten letters and visual art works, it may also take place during observing a curve that visually represents an abstract mathematical concept. When an observer processes a curve, a simulation of hand movements that produces that curve could take place. For example, when an observer looks at the hand-drawn curve of the mathematical function of *f(x) = −x*^2^, the hand movements that are involved in drawing this visual representation can be simulated (Figure 3).

In mathematics, many abstract concepts are transformed into a graphic representation such as a curve. This transformation significantly facilitates the process of understanding highly abstract mathematical concepts. In this process, the body movements that produce the graphic representations of abstract concepts can be simulated. Here, abstract mathematical concepts are grounded through their visual representations and the body movements that produce the visual representations. In this way, motor system can contribute to the grounding of abstract mathematical concepts. We will return to this in the following section when discussing visual representations of mathematical functions.

## Motor strength of visual representations

As mentioned, transforming one representation of a concept into another representation may allow us to use a wider range of cognitive and perceptual resources to understand that concept. The strong version of embodiment assumes that the motor system is involved in the understanding of those metaphors in which a concept is understood in terms of an action (Gallese and Lakoff, 2005). For example, during processing the metaphor *grasp an idea*, those regions of the motor system that are involved in actual doing of grasping are activated. This has been supported by the findings of some neuroimaging studies that have investigated the neural activations when people process metaphorical sentences describing abstract concepts in terms of body actions such as *grasp an idea* and *kick a habit* (e.g., Glenberg et al., 2008; Boulenger et al., 2009; Desai et al., 2011; Desai, 2021). Here, some sensory-motor resources are employed to understand a metaphor that describes a highly abstract event. This could happen in many linguistic metaphors in which an abstract event is understood in terms of a concept that has a high degree of sensory-motor strength. In these metaphors, sensory-motor resources may play a key role in the grounding of abstract concepts.

Fictive motion sentences are one group of metaphors in which a static concept is understood as a motion concept (Talmy, 1996). The sentence *The road passes through the desert* is a fictive motion sentence that describes the static concept of road as a moving object. The ways through which these sentences are processed have been the subject of a large number of works from a variety of perspectives (e.g., Lakoff and Núñez, 2000; Matlock et al., 2005; Richardson and Matlock, 2007; Blomberg and Zlatev, 2014; for a review, see Huette and Matlock, 2016). A number of behavioral studies have provided evidence that suggests understanding fictive motion sentences involves a mentally simulated motion (e.g., Boroditsky and Ramscar, 2002; Matlock, 2004, 2006; Núñez et al., 2006; Matlock et al., 2011). Reviewing a number of studies conducted on fictive motion sentences, Matlock (2010) suggests that people experience a fleeting sense of motion when processing such sentences. Also, the findings of a neuroimaging study suggest that understanding fictive motion sentences involves the activation of a region of the motor system that responds to perceived motion (Saygin et al., 2010).

**Figure 4:**
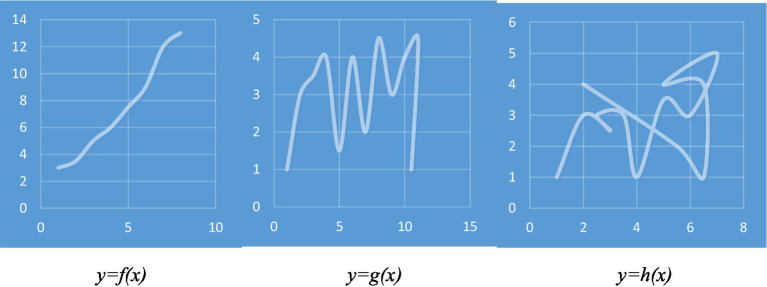
Three visual representations with different degrees of motor strength.

If fictive motion sentences create a sense of fleeting motion in the mind of the comprehender and activate the motor system, it may be said that visual tools that show such sentences can also have a similar effect on the comprehender. For example, when an observer looks at a photo that shows a road passing through a forest, s/he may experience a fleeting sense of motion. In the same way that the fictive motion sentence *The road passes through the forest* can be the cause of some degree of activation in the motor system, the experience of looking at the photo can be the cause of some degree of activation in the motor system. Here, the sensory (visual) and motor systems are employed to process the photo. The same thing can happen when the abstract representation of a mathematical entity is transformed into a visual representation. Visual representation of a function in a Cartesian plane is an example in which a mathematical entity is transformed into a visual representation. The visual representation of a function could be a curve in a Cartesian plane. The curve has a high degree of visual strength. Furthermore, it may have some degree of motor strength as well. Shape of a curve may be a key factor in the degree of activation of the motor system. The experience of observing some curves may involve a greater degree of motor activation compared to other curves. In other words, some curves may have a greater motor strength. In Figure 4, the visual representations of three functions in the Cartesian plane have been shown. Compared to visual representations of functions *g(x)* and *h(x)*, the visual representation of *f(x)* may have a greater degree of motor strength. Also, *g(x)* may have a greater degree of motor strength than *h(x)*.

**Figure 5:**
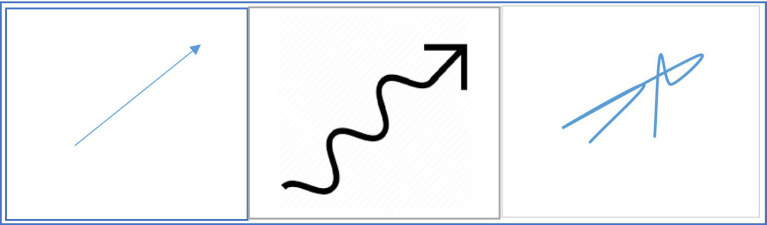
Different degrees of straightness and directedness.

A question that may be raised here is that what factors may affect degree of motor strength of the visual representation of a function. Findings of several studies can help us provide an answer for this question. In one of these studies, Pavan et al. (2011) found that photographs depicting different inferred speeds activate different speed-tuned neural populations. Williams and Wright (2009) examined the relationship between inferred speed in static images and patterns of activation in V5/MT. Their findings indicated a stronger activation for higher inferred speeds. Such findings suggest that some static images may have a stronger motor strength compared to other images. This could also be the case with visual representations of mathematical concepts such as curves. It is suggested that at least two features of a curve may be involved in determining the degree of motor strength of a curve (or a visual representation of a mathematical concept). The first feature is the degree of straightness and directedness. Those curves whose shapes are closer to a directed straight line (directed vector) may have a stronger motor strength compared to those curves whose shapes have a higher degree of in-directedness and curvature. Movement on a straight line with a certain direction is usually easier and all of us have the experience of moving with high speeds on a directed straight line. Also, the time that is needed to draw a directed straight line is usually shorter than the time that is needed to draw a curve with a high degree of curvature and in-directedness. It is easier for us to draw a directed straight line or a curve close to a straight line in a very short period of time, while it takes a longer period of time to draw a curve with a high degree of curvature and in-directedness. In fact, body movements that produce a directed straight line are usually faster and thus have a higher degree of motor strength. Those curves whose shapes are closer to a straight line may have a stronger motor strength because they are associated with movements with higher speeds and body movements that are produced in a shorter period of time. A comparison between the three visual representations in Figure 5 could make the point clearer. The left one is a straight vector with a clear direction. In many scientific discussions, such vectors are used to show the movements of objects. The middle one is not straight, but it has direction. When we say a curve has direction, it means that it does not have any complete backward point. It has some degree of motor strength, but its motor strength is weaker than the left one. The right one is not straight and does not have a certain direction. It has several backward points. Since backward points involve a moment of stop followed by a complete change of direction, they can reduce degree of motor strength. The right one has the weakest degree of motor strength.

The second feature that may be involved in determining degree of strength of a curve is the strength of association between shape of the curve and past experiences of the observer. For example, many of us may have had the experience of looking at moving objects along paths that were similar to the visual representation of the function *f(x)* in Figure 4. Therefore, when we look at this graph, we can expect a relatively strong degree of the motor system activation. On the other hand, we rarely have had the experience of looking at objects moving along a curve similar to visual representation of *h(x)*. Therefore, observing this curve may lead to a small degree of motor system activation or even no activation at all. In fact, it can be said that some kind of metonymic relationship is formed between the shapes of some curves and movement experiences. The strength of metonymic relationship is greater for some types of curves depending on past experiences of the observer. However, it should be noted that the way that a mathematical representation is viewed by a person depends on the person’s prior knowledge of the representation (e.g., Fletcher and Tobias, 2014; Van Gog, 2014; Schüler et al., 2015; Richter et al., 2018; Seufert, 2019; Vogt et al., 2020). Novice mathematics learners, adults, and experts view various representations differently. Prior mathematical knowledge may have a significant effect on reasoning and working with various representations. Therefore, for a certain individual, degree of motor strength of the visual representation of a function may be affected by her/his prior knowledge of that representation.

This association may be reliant on just pure shape of the curve. This is consistent with the findings of a drawing study conducted by Matlock (2006). Results of this study showed that people drew longer, straighter, thinner lines to visually describe abstract motion sentences that included fast manner verbs such as “race.” This is another indication that suggest some feature of the pure shape of a curve are associated with degree of motor strength of the curve. Therefore, it may be concluded that that pure shape of visual representation of a mathematical entity can be a key factor in the extent to which the motor system comes into play in the processing of that concept.

The idea that some visual representations or images have strong degrees of motor strength is supported even by some of our daily experiences. All of us have had the experience of having a fleeting sense of movement or a fleeting tendency to move when looking at some images. This phenomenon is so common in our daily experiences that most of the times we may not be aware of it. The image of a sprinter who is ready to run, a winding track, a map, or a vector are some examples of static images that may cause a sense of motion in the observer. On the other hand, the image of a closed door (preventing people from entering a place) or a blockage may create an opposite sense (being static and immobile). In fact, even very common daily experiences support this idea that some static visual representations or images cause a sense of motion in the observer, while others create a sense of being static. This phenomenon is not limited to our visual experiences. Even some of our aural experiences may cause a sense of motion in us. For example, some types of music cause a sense of movement in people (e.g., dance music, marching music), while others may create an opposite sense. In this case, it may be said that the first type of music has a stronger degree of motor strength.

The activation of the motor system may happen in mental imagery and in the absence of the object in the environment. Reviewing a number of works on mental imagery (e.g., Grush, 2004; Gallese, 2009; Delevoye-Turrell et al., 2010), Palmiero et al. (2019) suggest that spatial imagery may be reliant on perceptual and motor processes. They add that motor components may play a key role in mental imagery. The role of the motor system in the understanding of abstract concepts has been emphasized in a large number of works (e.g., Glenberg and Kaschak, 2002; Nathan and Walkington, 2017; Khatin-Zadeh et al., 2019, 2022a,b,c,d,e; Macedonia, 2019; Harpaintner et al., 2020). The following section discusses several examples in which mathematical entities are described in terms of visual representations and motion domains.

## Describing concepts in terms of visual representations and motion domains

When a function is transformed into a graph as its visual representation, the graph and motor systems come into play and help us to process that function in a more effective way. The graphical representation of a function reveals some features of the function that cannot be easily discovered through non-visual representations. The graphical representation of a function clearly shows where the local maximum and minimum points are, where the function is ascending, where it is descending, and many other features that are much more difficult to discover through non-visual representations. As mentioned in the second section, transforming one representation of a mathematical entity into a visual representation is in fact a mathematical metaphor. Mathematical metaphors allow us to employ a wider range of cognitive resources to ground and understand mathematical entities. Among these resources, the visual and motor systems were discussed.

**Figure 6:**
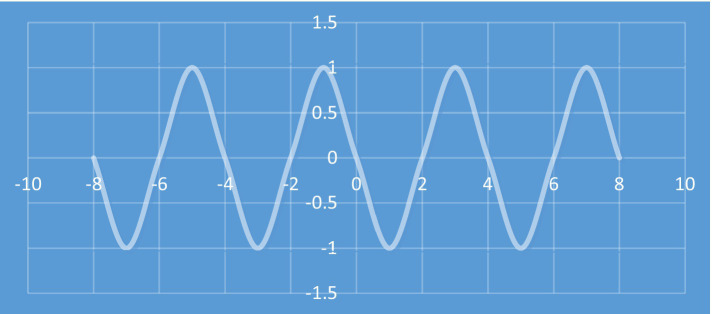
Visual representation of a sequence that oscillates between two values.

Lakoff and Núñez (2000) discussed a number of mathematical metaphors that are based on fictive motion. The metaphor *f(x) never goes beyond 1* is an example of such metaphors. When the algebraic representation of the function *y = f(x)* is transformed into a visual representation and the metaphor *f(x) never goes beyond 1* is used, the visual and motor systems are employed to process the behavior of this function. As mentioned, shape of the visual representation could play an important role in determining the extent to which the motor system is employed to process the behavior of this function. Núñez (2005, 2008) discussed several mathematical metaphors whose visual representations were expressed by gestures in a mathematics classroom. In one example, the visual representation of a sequence that oscillates between two values was described by gestures. The sequence included a series of numbers, which are highly abstract in nature. However, the visual representation of a sequence could be described in terms of a curve and a fictive motion in the form of an oscillation. Here, a mathematical concept was transformed into a visual representation shown by gestures. Both sensory and motor systems were employed to process the behavior of a highly abstract mathematical concept. The visual representation of such a sequence have been shown in Figure 6.

Since many of us have the experience of observing movement of objects along curves similar to the curve shown in Figure 4 [*f(x)*], it may be said that this curve has a great motor strength. Therefore, when we observe or even imagine this curve, our motor system may be activated. For more complex cases of curves, the past experiences of the comprehender may play an equally important role. If the comprehender has had the experience of observing complex movement of objects, that experience may help her/him to acquire a better understanding of those mathematical concepts whose visual and gestural representations are similar to those complex movements. This could have some implications for mathematics teaching classroom. We will return to this point in the following section.

**Figure 7:**
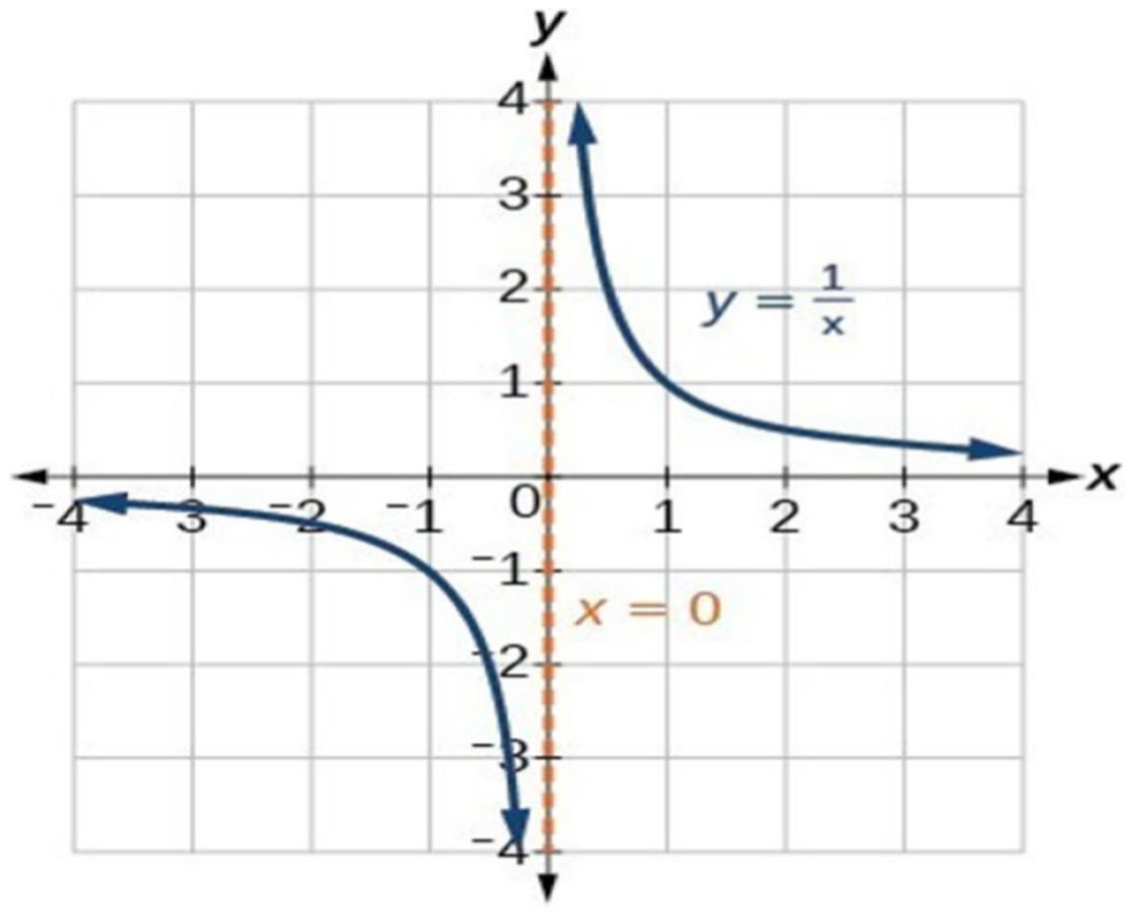
Visual representation of the function *y=* $\frac{1}{x}$.

A perhaps more complex example is the curve of a function that has an asymptote. The distance between the curve of a function and its asymptote approaches zero as one or both *x* and *y* coordinates tends to infinity. The asymptote of the function *y=* $\frac{1}{x}$ has been shown in Figure 7. The line *x = 0* is the asymptote of the curve of this function. As *x* approaches zero, the curve of the function approaches the line *x = 0*, and the distance between the curve and the line approaches zero. The behavior of this function and its asymptote line can be effectively understood through its visual representation and a fictive motion that shows the visual representation. In the definition of asymptote, the word ‘approach’ is used, which indicates this behavior of the function and its asymptote is understood in terms of a fictive motion.

The following section discusses pedagogical implications of using visual representations of mathematical entities and motion-based mathematical metaphors in mathematics teaching classroom.

## Pedagogical implications

Results of past studies have demonstrated how gestures could enhance the process of understanding mathematical concepts (e.g., Singer et al., 2008; Radford, 2009; Alibali and Nathan, 2012; Johnson-Glenberg and Megowan-Romanowicz, 2017; Nathan and Walkington, 2017; Yeo et al., 2017; Macedonia, 2019; Khatin-Zadeh et al., 2022f). It has been shown that describing concepts in terms of actions and gestures can facilitate speaking by organizing ideas into suitable units (Kita and Davies, 2009) and by lexical priming (Krauss et al., 2000). Also, gestures can contribute to the process of thinking by activating and maintaining mental images (Wesp et al., 2001). Result of a study conducted by Ravizza (2003) indicated that even meaningless gestures, which have no logical relationship with the meanings of words, may contribute to the process of retrieving lexical items in some cases. She attributed this to the activation of neural regions that are shared by speech and body movements.

In addition to these, priming mathematical concepts and ideas by their visual representations, by gestures that describe their visual representations, or by gestures that show a fictive motion related to their visual representations may facilitate the process of understanding mathematical concepts and ideas. This is particularly the case with those mathematical concepts and ideas which have components with great motor strength. Results of a study by Wilson and Gibbs (2007), which was conducted outside mathematics teaching, indicated that real and imagined gestures related to a metaphorical statement could facilitate comprehenders’ immediate comprehension of that metaphorical statement. In other words, real and imagined gestures related to a metaphorical statement could have a priming effect on the understanding of that metaphor. A similar thing can be applied in teaching and explaining mathematical ideas. Priming mathematical ideas by their visual and gestural representations could activate cognitive resources of the comprehender and enhance her/his understanding of those ideas. Gestural representations of concepts can be their literal or metaphorical representations. In explaining concepts related to vectors in the three-dimensional space, gestures could be used to literally represent concepts, as vectors are essentially movements in the three-dimensional space. However, in describing concepts such as limit and continuity, gestures can be used to metaphorically represent concepts. Although limit and continuity are algebraic and highly abstract concepts, they have visual and concrete representations. These two concepts can be effectively described through their visual and gestural representations. Using both literal and metaphorical gestures can be an effective technique in teaching mathematics as they help comprehenders employ a wider range of cognitive resources in the process of understanding.

## Summary

This article discussed the cognitive process of transforming one representation of mathematical entities into another representation. Various representations of the same mathematical entity have an underlying structural similarity. Therefore, parallel representations of a mathematical entity are inherently isomorphic with each other. That is, while parallel representations of a mathematical entity share an abstract underlying structure, they seem to be different in their concrete forms. The process of transforming one representation of a mathematical entity into another representation can be seen as a kind of metaphor. Mathematical metaphors allow us to understand and embody a difficult-to-understand mathematical entity in terms of an easy-to-understand entity. When one representation of a mathematical entity is transformed into another representation, more cognitive resources can come into play to understand the base entity in a more effective way.

Fictive motions are one special group of metaphors that are used to describe some important mathematical concepts such as limit and continuity. As mentioned, fictive motion sentences may create a sense of fleeting motion in the mind of the comprehender and activate the motor system. Therefore, visual tools that show such sentences can also have a similar effect on the comprehender, as visual tools can be seen as the visual translations of those fictive motion sentences. A similar process may take place when the abstract representation of a mathematical entity is transformed into a visual representation. Visual representation of a function in a Cartesian plane, which can be in the form of a curve, is an example in which a mathematical entity is transformed into a visual representation. The curve is a visual representation that may have some degree of motor strength. The experience of observing some curves may involve a greater degree of motor activation compared to other curves. In other words, some curves may have a greater motor strength. It was suggested that directed, straighter, thinner, longer curves have a higher degree of motor strength. These features of visual representation (curve) of a mathematical entity can enhance our understanding of that concept, as visual and motor resources are employed to process that concept. Another possible factor that determines motor strength of a curve is the strength of association between shape of the curve and past experiences of the observer. If an individual has had the repetitive experience of observing objects moving along a certain curve, the shape of the curve may have a great strength for her/him. Having more experience of observing moving objects along a certain curve could mean that the curve has a greater motor strength for the observer/comprehender. In fact, it can be said that some kind of metonymic relationship is formed between the shapes of some curves and movement experiences. The strength of metonymic relationship is greater for some types of curves depending on past experiences of the observer. Identifying other factors that may have a role in the degree of motor strength of visual representation is a question that can be investigated in future research.

## Data availability statement

The original contributions presented in the study are included in the article/supplementary material, further inquiries can be directed to the corresponding author.

## Author contributions

OK-Z wrote the first draft of this manuscript. DF and AB commented on it and revised it. All authors contributed to the article and approved the submitted version.

## Funding

This work was supported by the Norges Teknisk-Naturvitenskapelige Universitet.

## Conflict of interest

The authors declare that the research was conducted in the absence of any commercial or financial relationships that could be construed as a potential conflict of interest.

## Publisher’s note

All claims expressed in this article are solely those of the authors and do not necessarily represent those of their affiliated organizations, or those of the publisher, the editors and the reviewers. Any product that may be evaluated in this article, or claim that may be made by its manufacturer, is not guaranteed or endorsed by the publisher.
